# Supplementary material for: HIVprotI: an integrated web based platform for prediction and design of HIV proteins inhibitors
Source: J Cheminform. 2018 Mar 9;10:12. doi: 10.1186/s13321-018-0266-y (PMC5845081; doi:10.1186/s13321-018-0266-y)
Supplement: Supplementary file 2 — Additional file 2. Source code of HIVProtI web server. [file 13321_2018_266_MOESM2_ESM.zip › HIVprotI_Source-code/hivprotI/weka-3-6-6/doc/weka/classifiers/trees/Id3.html]

Id3


---


|  |  |  |
| --- | --- | --- |
| **Overview** \| **Package** \| **Class** \| **Tree** \| **Deprecated** \| **Index** \| **Help** \| **Weka's home** | |  |
| **PREV CLASS**   **NEXT CLASS** | **FRAMES**    **NO FRAMES**     **All Classes** |  |
| SUMMARY: NESTED \| FIELD \| CONSTR \| METHOD | DETAIL: FIELD \| CONSTR \| METHOD |  |


---


## weka.classifiers.trees Class Id3

```
java.lang.Object
  weka.classifiers.Classifier
      weka.classifiers.trees.Id3
```

**All Implemented Interfaces:**: java.io.Serializable, java.lang.Cloneable, Sourcable, CapabilitiesHandler, OptionHandler, RevisionHandler, TechnicalInformationHandler

---

``` public class Id3 extends Classifier implements TechnicalInformationHandler, Sourcable ```

Class for constructing an unpruned decision tree based on the ID3 algorithm. Can only deal with nominal attributes. No missing values allowed. Empty leaves may result in unclassified instances. For more information see:
